# Assessing Biological Response to Bevacizumab Using 18F-Fluoromisonidazole PET/MR Imaging in a Patient with Recurrent Anaplastic Astrocytoma

**DOI:** 10.1155/2015/731361

**Published:** 2015-02-22

**Authors:** Ramon F. Barajas, Miguel H. Pampaloni, Jennifer L. Clarke, Youngho Seo, Dragana Savic, Randall A. Hawkins, Spencer C. Behr, Susan M. Chang, Mitchel Berger, William P. Dillon, Soonmee Cha

**Affiliations:** ^1^Department of Radiology and Biomedical Imaging, University of California, San Francisco, 505 Parnassus Avenue, M-391, San Francisco, CA 94143-0628, USA; ^2^Department of Neurological Surgery, University of California, San Francisco, 505 Parnassus Avenue, Room 779 M, San Francisco, CA 94143-0112, USA; ^3^Department of Radiation Oncology, UCSF Long Hospital, 505 Parnassus Avenue, San Francisco, CA 94143-0226, USA

## Abstract

We present our initial experience in using single modality fluoromisonidazole (FMISO) PET/MR imaging to noninvasively evaluate the biological effects induced by bevacizumab therapy in a patient treated for recurrent high grade glioma. In this index patient, bevacizumab therapy resulted in the development of nonenhancing tumor characterized by reduced diffusion and markedly decreased FMISO uptake in the setting of maintained CBF and CBV. These observations suggest that the dynamic biological interplay between tissue hypoxia and vascular normalization occurring within treated recurrent high grade glioma can be captured utilizing FMISO PET/MR imaging.

## 1. Introduction

Glioma is the most common type of primary intra-axial brain tumor. Despite the aggressive combination of surgery, irradiation, and temozolomide based chemotherapy, median overall survival remains less than 15 months for high grade glioma [1]. Tumor recurrence remains the primary cause of mortality in this patient population with treatment options becoming limited and mostly ineffective when conventional therapy fails.

One of the pathologic hallmarks of malignant glioma recurrence is the presence of hypoxia. Hypoxia is a potent stimulator of vascular endothelial growth factor (VEGF) mediated angiogenesis [2]. In addition, hypoxia limits the efficacy of radiation therapy and chemotherapy. A better understanding of treatment related changes in regional tumor hypoxia may allow for the development of more effective patient-specific therapies. To this end, angiogenesis inhibitor therapy is currently administered to patients who have developed glioma recurrence. Bevacizumab, one of the most widely used angiogenesis inhibitors for glioma recurrence, is a recombinant humanized monoclonal antibody against VEGF. The goal of antiangiogenesis therapy is to disrupt and normalize neoplastic vascular formation in an attempt to prevent further tumor growth. Given the complex and synergistic interplay between hypoxia and angiogenesis, the pretherapeutic quantification of hypoxic tumor burden may be useful in risk stratification of patients who seek to undergo angiogenesis inhibitor therapy.

One noninvasive means of quantifying tissue hypoxia is through the use of 18F-fluoromisonidazole (FMISO) PET imaging. Several studies have validated FMISO uptake as a robust measure of tissue hypoxia [2–8]. Reduced preradiation hypoxic volume, as measured by FMISO PET, has been shown to be a good prognostic biomarker of clinical outcome in patients with treatment naïve glioblastoma [3]. Physiologic MR imaging methods such as dynamic susceptibility-weighted (DSC) perfusion and diffusion-weighted imaging (DWI) sequences may also contribute to the characterization of tumor biology and prognosis for patients with recurrent glioma. Combined, these PET/MR imaging sequences provide an opportunity to study the biological association among tumor hypoxia (by FMISO PET and DWI), cellularity (by DWI), and microvascularity (by DSC perfusion MRI). The combination of imaging data would be expected to be synergistic in delineating the mechanism between hypoxia and angiogenesis. As such, the assessment of hypoxic tumor burden prior to angiogenesis inhibitor therapy may provide useful information in predicting response for patients with recurrent glioma. However, there is currently a paucity of evidence regarding the role of hypoxic tumor burden in imaging response outcome following bevacizumab therapy.

In this case report we describe the FMISO PET/MR imaging changes that occurred in a patient with recurrent anaplastic astrocytoma undergoing bevacizumab therapy. Specifically, we demonstrate that, in this patient, bevacizumab therapy results in the progressive growth of recurrent nonenhancing cellular tumor manifested by reduced diffusion with decreased tumor hypoxia.

## 2. Case Presentation

A 65-year-old man with a history of left temporal lobe WHO grade III anaplastic astrocytoma presented with clinical and MR imaging evidence of tumor recurrence 9 months after initial diagnosis. At the time of diagnosis the tumor had molecular features consisting of isocitrate dehydrogenase 1 (IDH-1) wild type, epidermal growth factor receptor (EGFR) amplified, and O6-methylguanin-DNA-methyltransferase (MGMT) unmethylated. Following subtotal resection, the patient underwent external beam radiation therapy (6,000 cGy; 30 fractions) and concurrent temozolomide therapy for 8 months. Upon the diagnosis of glioma recurrence, the patient was enrolled in a clinical trial that included a bevacizumab treatment arm (10 mg/kg, 879 mg IV every two weeks).

One week prior to initiation of bevacizumab and following the fourth dose of therapy the patient underwent Institutional Review Board approved FMISO PET/MR imaging. Following informed written consent, the patient was administered 7 mCi FMISO intravenously. Simultaneous PET/MR imaging was performed on a 3T investigational general electric (GE) scanner 90 minutes following FMISO administration. Forty-minute PET emission imaging was performed with time of flight reconstruction. Attenuation correction utilized patient-specific T1-weighted map registered with segmented bone from a head CT image template. MR imaging sequences included an axial T1 precontrast (567/5 msec, TR/TE; 5/0 0 Slice Thickness/Skip (mm)), 3D CUBE FLAIR (5.34/163/2375 msec, TR/TE/TI; 1/0 mm), 3D T2 (3000/90 msec; 1/0 mm), axial diffusion weighted imaging (DWI) (8000/5 msec; 2/0 mm; 1,000 sec/mm^2^ B-value), dynamic susceptibility weighted contrast enhanced (DSC) perfusion imaging (1400/25 msec; 3/0 mm; 35° flip angle), and 3D gradient recalled T1-weighted postcontrast (34/3 msec; 1/0 mm) imaging.

Processing of DSC and DWI data was performed using a GE advantage workstation running Functool software v4.4. This allowed for the production of cerebral blood volume (CBV), cerebral blood flow (CBF), and apparent diffusion coefficient (ADC) physiologic maps. FMISO PET and MR sequences were coregistered using investigational GE PET/MR review v1.0 allowing for the production of regions of interest (ROIs) and generation of quantitative values. DSC and DWI maps were standardized to contralateral normal appearing white matter (NAWM) allowing for the production of relative values. Semiquantitative FMISO values were produced by standardization to ROIs placed on the right distal cervical internal carotid artery (tissue to blood; T/B value) and NAWM (rValue).

**Figure 1:**
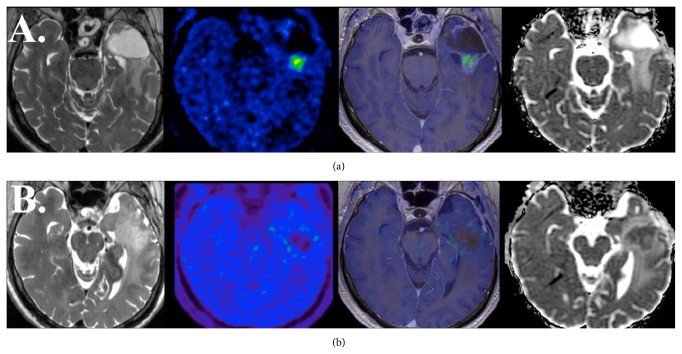
Single modality FMISO PET/MR imaging in a 65-year-old man with recurrent left temporal lobe WHO grade III anaplastic astrocytoma immediately prior to (a) and concurrent with bevacizumab therapy (b). Baseline, simultaneously obtained, axial T2 weighted (left), FMISO PET (middle left), fused FMISO PET and T1 weighted post contrast (middle right), and apparent diffusion coefficient (right) PET/MR imaging demonstrate recurrence of disease evidenced by contrast enhancing focus bordering the posterior margin of an anterior left temporal lobe resection cavity. This region demonstrates increased FMISO uptake with no evidence of associated reduced diffusion. Follow-up PET/MR imaging 10 weeks later (after 4 doses of bevacizumab) demonstrates the development of a nonenhancing T2 hyperintense mass about the posterior margin of the resection cavity that is associated with the development of reduced diffusion and diminished FMISO uptake.

Pre-bevacizumab FMISO PET/MR imaging demonstrated contrast enhancing recurrent disease about the posterior margin of the resection cavity associated with elevated CBV (CBV_min⁡_ 10.3, CBV_mean_ 59.2, and CBV_max⁡_ 154.8; rCBV_min⁡_ 1.25, rCBV_mean_ 1.84, and rCBV_max⁡_ 2.17) and CBF (CBF_min⁡_ 76.5, CBF_mean_ 397.2, and CBF_max⁡_ 773.8; rCBF_min⁡_ 1.55, rCBF_mean_ 1.23, and rCBV_max⁡_ 1.24). FMISO uptake within the contrast enhancing lesion was also elevated (T/B values, min 1.12, mean 1.43, and max 1.65; rValues, min 1.72, mean 2.11, and max 2.18) without associated reduced diffusion (ADC_min⁡_ 935, ADC_mean_ 1500, and ADC_max⁡_ 2690; rADC_min⁡_ 1.50, rADC_mean_ 1.86, and rADC_max⁡_ 2.53) (Figure 1(a)). FMISO PET/MR imaging 10 weeks after the initial exam (8 weeks following first dose of bevacizumab therapy, 4 doses administered as total) demonstrated a nonenhancing T2/FLAIR hyperintense mass about the posterior margin of the resection cavity that had developed reduced diffusion (ADC_min⁡_ 534, ADC_mean_ 707, and ADC_max⁡_ 1370; rADC_min⁡_ 0.77, rADC_mean_ 0.81, and rADC_max⁡_ 1.24) and decreased FMISO uptake (T/B values: min 0.46, mean 0.91, and max 1.43; rValues: min 0.49, mean 0.71, and max 1.65) (Figure 1(b)). The nonenhancing reduced diffusion lesion maintained CBV (CBV_min⁡_ 8.09, CBV_mean_ 50.8, and CBV_max⁡_ 190.6; rCBV_min⁡_ 1.09, rCBV_mean_ 0.99, and rCBV_max⁡_ 0.99) and CBF (CBF_min⁡_ 61.3, CBF_mean_ 295.4, and CBF_max⁡_ 761.5; rCBF_min⁡_ 1.28 rCBF_mean_ 1.18, and rCFV_max⁡_ 1.44). To date the patient continues bevacizumab therapy.

## 3. Discussion

We highlight our initial experience utilizing single modality FMISO PET/MR imaging to noninvasively evaluate the biological effects induced by bevacizumab therapy in patients treated for recurrent high grade glioma. In this index patient, bevacizumab therapy resulted in the development of nonenhancing tumor characterized by reduced diffusion and markedly decreased FMISO uptake in the setting of maintained CBF and CBV. These observations suggest that the dynamic biological interplay between tumor hypoxia and vascular normalization occurring within a patient with recurrent high grade glioma treated with bevacizumab can be captured utilizing FMISO PET/MR imaging.

The development of reduced diffusion concurrent with bevacizumab therapy is a well-established MR imaging feature, the etiology of which remains unclear [9–11]. Some investigations suggest that these lesions reflect aggressive hypercellular tumor growth. Other studies suggest that these lesions represent chronic hypoxia and necrotic tissue. In our patient, the region of reduced diffusion maintained baseline perfusion levels; however, it demonstrated FMISO uptake below the levels observed within normal appearing white matter. This finding seemed paradoxical as reduced diffusion would be expected in the setting of tissue hypoxia. In this case, we hypothesize that the emergence of a nonenhancing mass characterized by reduced diffusion and decreased FMISO uptake concurrent with bevacizumab therapy represents the presence of highly cellular recurrent glioma with transformed or normalized tumor microvasculature [12, 13].

The development of lesions with reduced diffusion concurrent with bevacizumab therapy has been shown to be prognostic of improved clinical outcomes [14]. The antagonism of VEGF receptors decreases the expression of abnormal microvascular morphology. This therapeutic effect is postulated to improve transport of oxygen and drug therapies [15]. As a result, one would presume to find improved efficacy of radiation therapy in patients manifesting reduced diffusion associated with decreased hypoxic burden following bevacizumab administration. It is important to highlight the point that DWI alone would not allow for the differentiation of hypoxic tissue from cellular recurrent tumor. Therefore, the combined examination of FMISO and DWI metrics in this patient population may allow for lesion biological stratification prior to the initiation of radiation therapy.

FMISO uptake has been previously characterized within newly diagnosed and recurrent glioma; however, to our knowledge, this is the first report of FMISO PET/MR imaging following bevacizumab therapy in humans with recurrent glioma. Cher et al. have previously investigated the aggressive biological features associated with FMISO uptake in 14 patients with treatment of naïve glioma [8]. Their investigation demonstrated a statistically significant association among FMISO uptake, vascular endothelial growth factor, and Ki-67 immunoreactivity suggesting that glioma hypoxia is biologically associated with angiogenesis and tumor cellular proliferation prior to therapeutic intervention. The association between aggressive biological features and FMISO uptake is consistent with the observations made in our patient's pretherapeutic PET/MR examination at the time of tumor recurrence: elevated FMISO uptake within the contrast enhancing region of recurrent tumor with elevated cerebral blood volume.

It is yet to be determined whether the loss of contrast enhancement within recurrent glioma following bevacizumab therapy should be considered a treatment success. According to the current response assessment in neurooncology (RANO) criteria the loss of contrast enhancement would be considered a complete response to therapy [16]. However, the clinical significance of this finding has yet to be definitively adjudicated. Furthermore, the development of reduced diffusion lesions following bevacizumab therapy, according to some investigations, would suggest the development of recurrent nonenhancing disease. The results of our study preliminarily indicate that these nonenhancing reduced diffusion lesions that develop following bevacizumab therapy are not the result of tissue hypoxia. Continued investigation into the prognostic utility of FMISO uptake following prolonged bevacizumab therapy still needs to be undertaken.

## 4. Conclusion

Our initial experience suggests that FMISO PET/MR imaging can noninvasively evaluate the dynamic effects induced by bevacizumab therapy in a patient with recurrent high grade glioma. We continue to enroll patients in this study to further adjudicate the biological effects of bevacizumab therapy on tumor hypoxic burden.
